# Man is a “Rope” Stretched Between Virosphere and Humanoid Robots: On the Urgent Need of an Ethical Code for Ecosystem Survival

**DOI:** 10.1007/s10699-021-09796-z

**Published:** 2021-06-17

**Authors:** Luigi F. Agnati, Deanna Anderlini, Diego Guidolin, Manuela Marcoli, Guido Maura

**Affiliations:** 1 Department of Neuroscience, Karolinska Institutet, Stockholm, Sweden; 2 Department of Biomedical Sciences, University of Modena and Reggio Emilia, Modena, Italy; 3 Centre for Sensorimotor Performance, The University of Queensland, Brisbane, Australia; 4 Department of Neuroscience, University of Padova, Padova, Italy; 5 Department of Pharmacy and Center of Excellence for Biomedical Research, University of Genova, GENOVA, Italy

## Abstract

In this paper we compare the strategies applied by two successful biological components of the ecosystem, the viruses and the human beings, to interact with the environment. Viruses have had and still exert deep and vast actions on the ecosystem especially at the genome level of most of its biotic components. We discuss on the importance of the human being as contraptions maker in particular of robots, hence of machines capable of automatically carrying out complex series of actions. Beside the relevance of designing and assembling these contraptions, it is of basic importance the goal for which they are assembled and future scenarios of their possible impact on the ecosystem. We can’t procrastinate the development and implementation of a highly inspired and stringent “ethical code” for human beings and humanoid robots because it will be a crucial aspect for the wellbeing of the mankind and of the entire ecosystem.

## General Premise

The history of ecosystem is based on the concept of “evolution” that usually implies some sort of “progress” towards life-forms more adapted to the environmental challenges. As a result, a “staircase” with homo sapiens at the top is a usual metaphorical representation of the concept. Homo sapiens’ unique cognitive capabilities are considered not only as the best but also as the unique criterium to evaluate the rank order of the different species in the ecosystem panorama.

In the present paper less self-referential and more wide-range criteria are proposed not to suggest a different “staircase” but rather to consider the impact of the many different life-forms^1^ on the ecosystem and at which extent they are capable of adaptation to the ecosystem changes. Accordingly, the following two criteria are suggested:

- (a) The life-form’s capability to impact the biotic and abiotic components of the ecosystem;
- (b) The life-form’s capacity of diffusion in the ecosystem and of mutation to adapt to ecosystem changes/challenges.

The present paper focuses on two biotic components of the ecosystem, namely the virosphere (Villarreal, 2016) and the human beings, since they share the capability to markedly modify the biotic components of the ecosystem and a high capacity of diffusion in the environment. It should be noted that life-forms with a high impact on the ecosystem, especially if characterized by a vast diffusion, may produce significant changes in the entire ecosystem.

The first identification of viruses in the nineteenth century saw them as “pathogens” therefore it was important to study their pathogenicity and replication’s mechanisms to prevent and fight the diseases caused by them (Watanabe & Kawaoka, 2020). Nowadays, it is time to adopt a new perspective in virology because the huge abundance of viruses in nature together with their prowess to move among hosts make them an integral component of the ecosystem (French & Holmes, 2020). Viruses, certainly, impact on ecosystem health, resilience, and function, but, in turn, host ecology impacts viral abundance and diversity. In some circumstances, viral infections have positive impacts either on the evolution of their hosts (Mi et al., 2000) or on their biological function (Herbein, 2018; Kortright et al., 2019). It has been estimated that about 8% of the human genome is derived from viruses (Consortium, 2001). Investigations in the fields of metagenomics and metaviromics have resulted in the identification of an enormous number of viral sequences to form the so-called virosphere. Even more interesting, viral sequences have been found in different regions of the globe confirming viral ubiquity (Kristensen et al., 2010; Suttle, 2005). Still, we agree with Rodriguez and collaborators that although there has been a big step forward in the identification of new viruses and their interaction with the hosts, we don’t know yet how this “interactive network” is connected (Rodrigues et al., 2017).

Viruses, therefore, have many of the potential winning characteristics in the evolutionary competition. Their vast spatial diffusion, their replication in a host mechanism allowing a low energy expense, and their high capability of adaptation outline a biological strategy of significant impact on the environment, since they can modulate almost the entire biotic component of the ecosystem (Margulis, 1993, 2008) from a genetic standpoint. Thus, they likely had a central role during evolution (Chisholm et al., 2019; Simonson et al., 2010; Villarreal, 2016) and they certainly play a role today.

In terms of the produced impact, however, human beings appear to represent a further step. They, indeed, are contraptions makers and, through these instruments, they can not only create survival niches, in order to adapt to ecosystem challenges, but also markedly increase their diffusion and deeply change the environment. As far as the development of human-made contraptions is concerned, in the near future they will be likely more and more endowed with Artificial Intelligence (AI) (Muzyka, 2020) and social cognition leading to the development of “humanoid robots” with potentially significant consequences on the equilibrium of the ecosystem. Therefore, while viruses, like any other living^2^ thing, simply adapt in order to spread and ensure their continued existence (although in that process, they could, to a certain degree, damage biological ecosystem), humans exert dominance over the ecosystem, trying to adapt the environment to themselves potentially harming the ecosystem "beyond reparable" (Barnosky et al., 2011; Diamond, 2005; Lorenz, 1973). This human attitude could be likely linked to the process used by the human brain to acquire knowledge about the world. As pointed out by Zeki (Zeki, 2002), such a process is basically a diffuse and sophisticated process of abstraction that extracts specific attributes from sensory modalities (Agnati, Guidolin, et al., 2012). This process of generalization is a very efficient way in which a finite mind grasps the infinity of particulars and also frees the brain from total dependence upon a memory system (Zeki, 2002). However, there is also a price to be paid for this. As suggested by philosophical (Schopenhauer, 1970; Seneca & Mutschler, 1969) and literature (Leopardi, 2011;3 Pirandello, 2013)^4^ works, it can lead to a sort of “unsatisfaction”^5^ induced by the perceived distance between the real experience and the rich spectrum of abstract models, ideas and concepts generated by the human brain. One way to obtain that satisfaction would be to “download” (Zeki, 2002) the ideas formed in the brain into the external world. In this respect, as mentioned above, in a famous Lecture the Nobel Prize Konrad Lorenz mentioned the possibility that the mankind unconsult actions (“Todsünden” or deadly sins) could lead to an overexploitation of the ecosystem (Lorenz, 1973). Thus, he required human beings to use a more conscious judgment to adjust their interactions with all the biotic and abiotic components of the ecosystem. Such a judgement should be based on an “ethical code” that considers the human being and the ecosystem as a unified “survival unit” (Bateson, 1972b; Guidolin, Marcoli, et al., 2019). The search for an ethical code is basically possible just because of the human being cognitive capabilities and it is, in several instances, one of the major accomplishments of philosophies and religions. Actually, the very attempt to define ethical codes overcoming the transient fate of the humans and of the single human communities is the mark of mankind since it is found in all cultures and in its entire History (Agnati et al., 2007, 2017a; Bloch, 2008). It has been proposed that this peculiarity results from the extraordinary potentialities of human being “inner-speech” that accompanies and informs human being during his entire life (Agnati, Barlow, et al., 2012). This aspect has been underlined by neuroscientists (Morin, 2009; Morin & Everett, 1990) and by great scholars (Leopardi, 1827). The term “inner speech” doesn’t refer only to the mental use of language, or simulation of speaking that often occurs consciously in the absence of any overt articulation. It mainly refers to a cognitive process (Langland‐Hassan, 2020) and there is a close connection between inner speech and perception/motor control with “higher” or “abstract” forms of thought (Clark, 1998; Dove, 2014). It is now considered a multi-component process with dissociable parts that are under research to understand the development and maintenance of human self-awareness (Morin, 2018) and to explain psychiatric phenomena such as auditory verbal hallucinations.

**Fig. 1:**
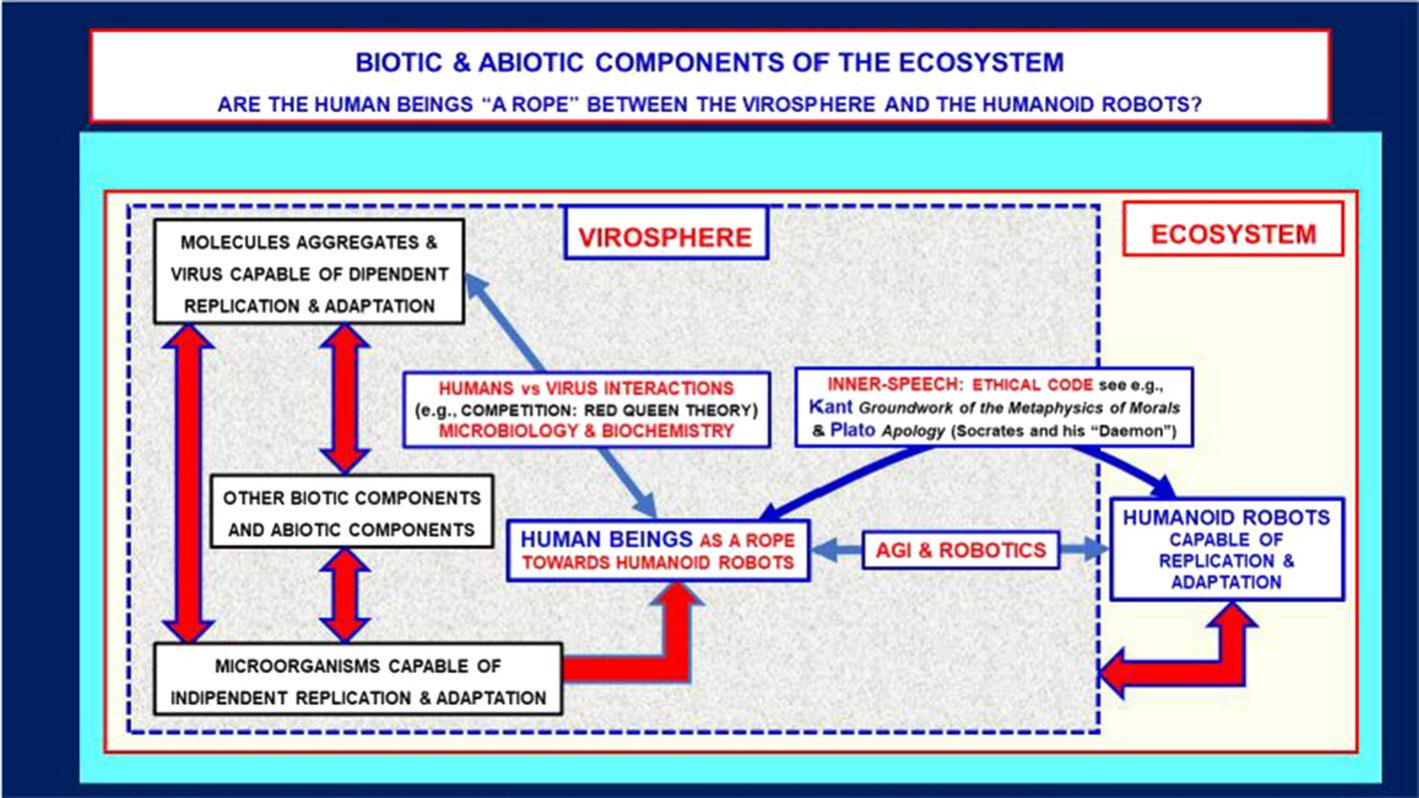
Schematic representation of the evolutionary course from the virosphere to the humanoid robots via the human beings. The virosphere occupies the main space of the ecosystem and viruses have several survival niches in it. The human beings have multiple possibilities of interactions with viruses such as symbiotic, competitive or indifferent coexistence. In the case of competition, it has been introduced the “Red Queen Theory” to describe the dynamic equilibrium that may be reached (Bonachela et al., 2017, Van Valen, 1974)

In this context, it is of particular importance that the ethical code would also define the designing and assembling of man-made contraptions, in particular those derived from the development of AI, such as humanoid robots (see Fig. 1). This aspect will put forward significant challenges: should AI contraptions be equipped with human ethics or should we allow AI to develop its own ethical code? From one side human ethics forms a heterogeneous set of moral values that may not be universally shared but rather be clustered across different groups of people. Thus, programming humanoid robots with human ethics means that it is some human group's ethics, which may not necessarily be approved by another group. On the other hand, allowing artificial intelligence to develop ethics from the scratch might end up humanoid robot behavior being more similar to that of viruses rather than that of human beings. In this respect, the possible development of an ethical code specific for AI contraptions might provide an answer to the question.

In the present discussion, therefore, as a starting point for a debate on hypothetical future scenarios, we will try to compare the human impact on the evolutionary course of the ecosystem with the one exerted by another impacting biological entity like viruses. In this respect, as mentioned before, humans exhibit the specific capability to develop contraptions. Thus, possible future outcomes of such a skill, namely artificial intelligence and humanoid robots, will be also briefly discussed, since they may acquire increasing importance to the point that they may reach a leading role in the ecosystem equilibrium, stressing the need for a new ethical framework.

## A Hypothesis on the Possible Evolutionary Course of the Ecosystem

In accord to the main points of the General Premise, the following hypothesis is put forward. In an evolutive context, the human beings can be considered a “bridge” connecting the most primitive strategies and driving the relationship of widespread forms of life and/or quasi-life (general term to indicate e.g., auto-replicants organic molecules) with their environment and a strategy based on contraptions which in the future may be endowed with artificial intelligence (“humanoid robots”). This made several authors believe they could become leaders capable of mastering the ecosystem in the future (Fig. 1). As in the title of the present paper, we paraphrase a famous sentence by Nietzsche^6^ in his masterpiece *“Thus Spoke Zarathustra”*:

*“The human being is a rope stretched between the virosphere and the Humanoid Robots (*Nietzsche & Common, 1950*).”* Homo sapiens is seen as a crucial “rope” in the evolutionary history of the so-called biotic and abiotic components of the ecosystem since he not only interacts and modulates but also greatly modifies both (Agnati et al., 2018; Guidolin, Marcoli, et al., 2019).

As well as other living beings of the ecosystem, human being creates ecological niches that increase his chances of survival and reproduction. In these circumstances, he should have a particular care of the ecosystem services (Coutts & Hahn, 2015; Guidolin, Anderlini, et al., 2019) like the water. Water is produced during the hydrological cycle that is strictly linked to plant and animal materials as food used by heterotrophic organisms (humans).

Moreover, humans have now become capable of reshaping virtually the entire ecosystem through their complex contraptions that have improved to the point of showing “intelligent” ways to fulfil difficult tasks. Such humanoid robots can perceive the key features of the environment, to imagine future scenarios, take actions to maximize their chance of achieving the hunted and predicted goals (Howard et al., 2019; Sandini et al., 2018) and likely they will play a leading role in the final phase of the ecosystem’s evolution.^7^ All this can happen only if, in the meantime, the human being will not cause an irreversible over-exploitation of the ecosystem ending with the sixth mass extinction (Agnati et al., 2018; Barnosky et al., 2011; Diamond, 2005; Lorenz, 1973).

If humanoid robots will be the endpoint of the evolutionary path, their “Demiurge” must also carefully define their “ethical code”. As pointed out above, robots’ actions should follow an ethical code that take into consideration not only human beings but rather the entire ecosystem (Westerlund, 2020).

## On Some Potential Winning Features in the Evolutionary Competition and the Peculiar Characteristics of the Viruses that Contributed to the Progressive Sophistication of the Biotic Components of the Ecosystem

As pointed out earlier, viruses have many of the potential winning characteristics in the evolutionary competition:

1. Spatial vast diffusion since they can create their niches in both abiotic and biotic components of the ecosystem (Coutts & Hahn, 2015);
2. High rate diffusion in the environment taking advantage of biotic (e.g., animals that migrate and especially now fast and far travelling human beings) and abiotic (e.g., sea tides and river flows) components of the ecosystem (Guidolin, Anderlini, et al., 2019);
3. Replication in a host, with the consequent great advantage of a low energy expense but the disadvantage of the need to find a suitable host (Moreno-Altamirano et al., 2019);
4. High capability of adaptation with possible symbiotic, indifferent, conflictual interactions with the host (Ayansola et al., 2019; Bahir et al., 2009);
5. High capability of mutations that allows them to spill over from one species to another one (Carrasco-Hernandez et al., 2017; Sanjuán & Domingo-Calap, 2016).

Since they can genetically modulate almost the entire biotic component of the ecosystem (Ozili & Arun, 2020; Plowright et al., 2017), they likely had and still have a central part during evolution (Sanjuán & Domingo-Calap, 2016; Villarreal, 2016; Villarreal & Witzany, 2010).

The balance of the several advantages and few disadvantages of survival and invasion of the ecosystem gives viruses an important role in the functional and structural organization of the ecosystem itself (Villarreal & Witzany, 2010). Viruses, like any other living thing, adapt in order to spread and ensure their continued existence.

The prevalent negative view on virus invasion of the biotic components of the ecosystem is understandable especially today since, via cross-species transmission (host jumping or spill over), viral pandemic^8^ diseases have imposed a major healthy and economic burden to our human societies (Ozili & Arun, 2020; Plowright et al., 2017). However, as underlined by French and Holmes (French & Holmes, 2019, 2020), viruses are not only pathogens responsible for potentially devastating diseases but also symbiotic components in ways that are beneficial in favouring adaptation processes of their hosts (Enard, 2016). Furthermore, it should also be remembered that the total number of viruses is two or even three times the number of existing cells in our ecosystem. Not least, hosts are infected by multiple viruses and sometimes at high abundance, but relatively few are clearly associated with disease (Shi et al., 2018; Zhang et al., 2018) while viruses have greatly contributed to the evolution of human being (French & Holmes, 2020). In support to such a view, it should be mentioned that a significant percentage of the human genome is composed of sequences related to viruses (Lander et al., 2001; Moelling & Broecker, 2019) where a complex interplay occurs with beneficial and dangerous aspects. Hitherto, in 1973 Leigh Van Valen proposed the “Red Queen Theory” (Bonachela et al., 2017; Clarke et al., 1994, Van Valen, 1974) to describe some aspects of the complex interactions, to estimate the “evolutionary potential” of pathogens and the capability of a pathogen to adapt to shifting environments (Geoghegan et al., 2016; Obbard & Dudas, 2014). One important point is that mutation for viruses, while it supplies a pool of raw genetic material, it can also generates many unfit mutants with a decrease of the virus’ evolutionary potential (Chisholm et al., 2019).

Another major aspect of viral evolution is due to their capability of collecting inherited traits changes during their lifetime. This feature facilitates the adaptation to environmental fluctuations and the overcoming the host immune response (Chisholm et al., 2019).

Summing up, viruses struggle in a usually balanced fight with the host immune response and seldom they defeat them resulting in an epidemic. As a consequence, either the host would adapt to the virus infection or will become extinct. However, such an adaptation process may contribute to evolution of the virus and the host. It becomes clear how viruses’ impact on the adaptation and genetic changes of human and other species are crucial components of the ecosystem (Mirahmadizadeh et al., 2019).

## Peculiar Characteristics of the Human Beings: in Respect of the Creation of Survival Niches and Contraptions Capable of Human-like Interactions with the Biotic/Abiotic Components of the Ecosystem

When compared to the biological successful strategy exhibited by viruses in their relationship with the environment, humans show some similarity. A human being can survive in a great many different physical environments as long as the chemical-physical conditions are such that he can maintain the physical homeostasis of his body thanks to multiple feedback mechanisms (Agnati et al., 2017b). Therefore, the human being continuously interacts with the survival unit to which he belongs: the biotic and abiotic components of the ecosystem and the supra-systems (that is the socio-cultural context) in which he lives (Agnati et al., 2017b). These complex interactions have cascade effects on his feeling of well-being, and hence on his psychic homeostasis that can modulate the physical homeostatic responses. A crucial feature of the adaptation is the possibility to trigger allostatic controls especially in response to psychic challenges (Agnati et al., 2017b; Sterling, 2012).

Natural selection operates on the human beings and the other components of the ecosystem, and adaptive evolutionary processes are such because they allow more efficient interactions between biotic and/or abiotic components of the unit of survival (humans and his ecosystem) (Bateson, 1972b). This occurs by means of appropriate changes in some of their features favouring survival and reproduction for human beings.

The human strategy to interact with the ecosystem exhibits also a key difference when compared to the one evolved in other life forms. Human being’s actions on the ecosystem go beyond a purely biological interaction of creating survival niches. Being increasingly based on the development and use of contraptions, it leads not only to the pollution, overexploitation and deep modification of natural environments, but also to the production of new species thanks to biotechnologies (Agnati et al., 2018) and as a result, deeply and often irreversibly altering the entire ecosystem equilibrium. In this respect, epidemiological data should be carefully considered too since they clearly demonstrate that alterations of the ecosystem equilibrium favour, inter alia, the emergence and diffusion of dangerous viruses (Agnati et al., 2017a; Guidolin, Anderlini, et al., 2019).

As far as the development of human-made contraptions is concerned, in the near future ‘humanoid robots” will be more and more endowed with Artificial General Intelligence (AGI) (Muzyka, 2020) and social cognition, they will likely evolve to interact with humans as cooperating robots or ‘‘cobots’’ and they will be able to perform complex tasks in an independent way (El Zaatari et al., 2019). The thought of robots with cognitive capabilities is a daunting one, characterized by scientific as well as technological aspects that are far from being sufficiently understood especially in the case of symbiotic robotic agents (Sandini et al., 2018; Sheridan, 2020). In our opinion, the main challenge is the ethical code that should guide those designing/producing humanoid robots and be embodied in the robots to make sure robots will always act with respect for the human beings along with the ecosystem (Bateson, 1972b; Fiske et al., 2019).

Thus, critical evaluations of ethical codes should be discussed first because they are even more important than AGI progresses and instrumental capabilities in creating these sophisticated contraptions. This long-term code will last much longer than building robots that can evolve in real hardware (Jelisavcic et al., 2017).

## The Final Step of the Evolutionary Course of the Ecosystem: the likely Leading Role of Highly Sophisticated Humanoid Robots

A positive outcome of the robot technology is the cutting of the burden on medical professionals that is rapidly increasing in the aged western society. “Social robots” are not just contraptions for physical manipulation in industries but rather robots effective for the human multiple needs (Sheridan, 2020). The field of “Social Robotics” is gaining relevance in the organisation of the health assistance since social robots serve a person in a caring interaction rather than to perform only mechanical tasks (Sheridan, 2020). In particular, healthcare robots will be highly useful in the assistance of aged people but even more in the light of possible pandemic infections since they can take care of infected people without risks. These more complex capabilities imply a basic aspect of the human being interactions with robots that is how to treat them as social entities. In the near future, humanoid robots will be endowed with the ability to perceive and to be perceived as intentional beings, hence as beings with a “mind” (Wiese et al., 2017). Social interactions will be possible based on the ability of humans and humanoid robots to understand actions and intentions of each others (Wiese et al., 2017).

**Fig. 2:**
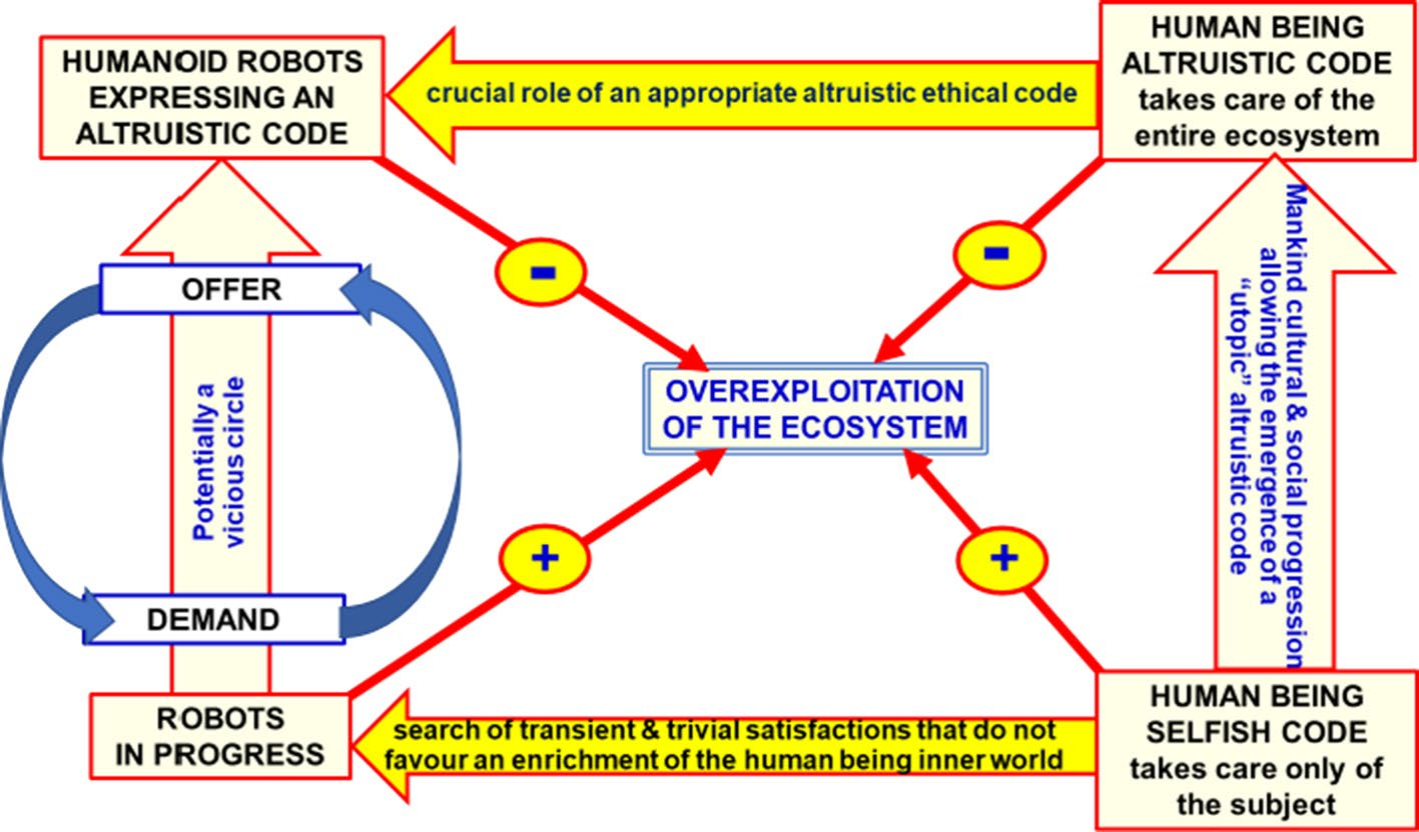
On the left: Schematic representation of the progress of robots that is mainly triggered by a vicious circle between “offer vs demand” since the human beings often are moved by an unexhausted search of “empty and ephemeral pleasures” that increases the demand of superfluous consumer goods. Such insatiable request to satisfy unnecessary needs is made possible by a selfish ethical code. On the right: Schematic representation of the desired emergence of “utopic” human beings governed by an altruistic ethical code

One of the main topics of the present paper are the implications of the development of robots capable of overcoming human cognitive abilities and to take decisions in accord to their own ethical codes (Figs. 1 and 2). This aspect is crucial and should be discussed in depth as soon as possible (Joerin et al., 2020). Perhaps, some implications can’t be completely and safely solved now. Certainly, the human history is full of unethical decisions and actions against the mankind and the ecosystem causing destructions and bereavements (Agnati et al., 2017a). Consequently, humanoid robots capable of a decisional power impacting the present and the future of the entire ecosystem should be endowed with an even higher and more stringent ethical code. “Super-human ethical robots” have been a dream not only of scientists but of great writers as Giacomo Leopardi (Leopardi, 2011). Scholars have foreseen possible advancements in evolution thanks to ethical robots capable of self-awareness, of taking ethical decisions, of performing actions to guarantee the best conditions for the entire ecosystem, ergo for all the survival units.

It is now clear that the ethics of AGI and artificial intelligent beings is an issue of basic importance for the future of mankind (Fjeld et al., 2020; Jobin et al., 2019). This could be the final endeavour of the moral behaviour of humans if they are fully aware that they have to design, construct, use AGI devices according to high ethical principles. A philosophical and jurisdictional fundamental effort is required to define ethical principles binding human beings and humanoid robots’ behaviours (Beduschi, 2020). This extremely complex issue has been already pointed out in a popular form by the science fiction writer Isaac Asimov who suggested the “Three Laws of Robotics” (known as Asimov's Laws) (Asimov, 1950):

- 1st Law: A robot may not injure a human being or, through inaction, allow a human being to come to harm.
- 2nd Law: A robot must obey the orders given it by human beings except where such orders would conflict with the First Law.
- 3rd Law: A robot must protect its own existence as long as such protection does not conflict with the First or Second Law.

Obviously, the problem is much more intricated because the acquisition of self-awareness and the increased cognitive abilities will favour the compulsory propensity to selfishness (Lee, 2020). To understand the risky complexity of the problem, it is enough to reflect that what actually is needed is for humans and robots to have a deep altruistic feeling, a feeling not only during their interactions but also during their interactions with the entire ecosystem.

Mankind is becoming aware of the existential risks arose from progresses in AGI and with them, of the paramount importance of finding a solution for the ethical question. This aspect has been pointed out by several authors (Karnofsky, 2016; Naudé & Dimitri, 2019) and popularised by Stephen Hawking and Elon Musk (Sparkes, 2015). They warned that a not wisely controlled AGI could someday become the dominant form of intelligence on Earth with “intelligent” robots uprising, enslaving mankind and even causing mankind extinction or a global catastrophe if convenient for robots themselves. Human beings can’t afford to have simply a Darwinian view of “survival of the fittest”.

In our opinion, as far as his own behaviour and the embodied ethical code of cobots, mankind should rather follow the often-cited Kantian approach:

*Act in such a way that you treat humanity, whether in your own person or in the person of any other, never merely as a means to an end, but always at the same time as an end* (Kant, 1994)

but this ethical dictum should be amplified with Bateson’s proposal “The unit of survival is organism plus environment” (Bateson, 1972a).

In other words, human being should *“Act in such a way to treat each component of the entire ecosystem never merely as a means to an end, but always at the same time as an end*.”

Hence, the inclusion of both human beings and ecosystem wellbeing should be the essential feature in view of their interdependence for their worthwhile survivals (Ford et al., 2015; Guidolin, Anderlini, et al., 2019).

Thus, mankind has to solve an ethical problem much more complex than an AGI problem and there are serious doubts, in view of the past human being history, that mankind is skilled for such an outstanding ethical task (Buruk et al., 2020).

## Concluding remarks: Crucial Relevance of a Highly Inspired and Stringent “Ethical Code” for the Ecosystem Survival

*To be, or not to be, that is the question* The human being has to face crucial tasks for the safety and positive future of his survival unit (Agnati et al., 2018; Bateson, 1972b) which is to be the “demiurge endowed with a Kantian ethics considered in an ecosystem to be carefully respected”, inter alia, connecting the two sides of the bridge: the virosphere and the humanoid robots.

In regard to the virosphere, the human beings can work to keep the viruses’ diffusion and mutation under a reasonable control. Important aspects of these scientific efforts have been mentioned in an interesting paper (Moratorio & Vignuzzi, 2018): “*What methods are available to follow viral evolution? Is it possible to predict virus evolution? Can we use this knowledge to drive deadly pathogens towards evolution's dead end, to extinction?*”. It seems possible to describe crucial aspects of virus evolution and by means of mathematical modelling, bioinformatics, and experimental evolution to potentially predict virus evolution in a proximate future as well as to investigate the most effective antiviral approaches to alter these trajectories.

Pertaining to the humanoid robots, it will be of fundamental importance not simply the human control of their impact on the human well-being but the “embodiment” of an ethical code in the humanoid robots that by default make them interacting appropriately with the entire ecosystem, where default is the essential key. A discussion about such an ethical code can start with the words of the great philosopher, Plato. In the “Apology of Socrates” where a peculiar aspect of Socrates “inner-speech” is described (Reale, 2001), Plato stated: “… Socrates mentions that in the processes of taking substantial decisions he is guided by a *daemon*, a kind of divine spirit-oracle, that takes the form of an inner voice or non-vocal compulsion. The guide never tells Socrates what to do. It only indicates when Socrates is *not* to do something.”

This fascinating assertion is a powerful clue to fully enjoy the beauty of a famous sentence by Kant:

Two things awe me most, the starry sky above me and the moral law within me.

Our last comment, that is also the conclusion of the present paper reads:

The fate of the entire ecosystem will depend on the human being ethical code.

On the other side, human beings create humanoid robots that can reach such a level of complexity and autonomy to become in a future the leaders of the ecosystem.

Thus, of paramount importance will be the ethical code in designing and producing such devices and even more to make them in such a way they will follow an ethical code that respect the human beings as well as the entire ecosystem. This aspect is discussed in the paper referring the thinking of great philosophers, in particular Plato and Kant.

## Footnotes

1 As Kejnovskya and Trifonov underline recent discoveries have established a background for embracing viruses and other replicating genetic entities into a more comprehensive tree of life (Kejnovskya E, Trifonov EN. Mobile Genetic Elements 2016, Vol. 6(2)).

2 In biology it is common to distinguish between living and inanimate matter by the ability to synthesize proteins, repair itself (autopoiesis) and eventually replicate autonomously. Some authors consider the giant viruses as a missing link between the two, because they harbour “almost” the protein synthesis apparatus. Thus, giant viruses may indicate that there is no sharp border between living and non-living entities but an evolutionary continuum. (Moelling K and Broecker F (2019) Viruses and Evolution – Viruses First? A Personal Perspective. Front. Microbiol. 10:523–533).

3 “*Ho anch’io, inesorabilmente radicata nel più profondo del mio essere, la stessa malattia dell’amico mio. La quale, a mio credere, dimostra nel modo più chiaro, che tutto quello che avviene, forse avviene perché la terra non è fatta tanto per gli uomini, quanto per le bestie. Perché le bestie hanno in sé da natura solo quel tanto che loro basta ed è necessario per vivere nelle condizioni, a cui furono, ciascuna secondo la propria specie, ordinate; laddove gli uomini hanno in sé un superfluo, che di continuo inutilmente li tormenta, non facendoli mai paghi di nessuna condizione e sempre lasciandoli incerti del loro destino. Superfluo inesplicabile, chi per darsi uno sfogo crea nella natura un mondo fittizio, che ha senso e valore soltanto per essi, ma di cui essi medesimi non sanno e non possono mai contentarsi.”* A malady which, to my mind, proves in the clearest manner that everything that happens happens probably because the earth was made not so much for mankind as for the animals. Because animals have in themselves by nature only so much as suffices them and is necessary for them to live in the conditions to which they were, each after its kind, ordained; whereas men have in them a superfluity which constantly and vainly torments them, never making them satisfied with any conditions, and always leaving them uncertain of their destiny. An inexplicable superfluity, which, to afford itself an outlet, creates in nature an artificial world, a world that has a meaning and value for them alone, and yet one with which they themselves cannot ever be content,…

4 “Non dubito però, che col tempo si arriverà a sopprimermi. La macchinetta – anche questa macchinetta, come le altre macchinette – girerà da sé. Ma cosa poi farà l’uomo quando tutte le macchinette gireranno da sé, questo, caro signore, resta ancora da vedere.” I have no doubt, however, that in time, Sir, they will succeed in eliminating me. The machine--this machine too, like all the other machines--will go by itself. But what mankind will do then, after all the machines have been taught to go by themselves, that, my dear Sir, still remains to be seen."

5 “*La soledad no es vivir solo, la soledad es no ser capaz de hacer compañía a alguien o algo que esta’ en nosostros.”…* loneliness is not living alone, loneliness is the inability to keep someone or something within us company.” Saramago, J., 1992. The Year of the Death of Ricardo Reis. 1984. *Trans. Giovanni Pontiero. New York: Harvest*.

6 Friedrich Nietzsche: *Thus Spoke Zarathustra* I love him who labours and invents, that he may build the house for the Superman, and prepare for him earth, animal, and plant: for thus seeks he his own down-going.

7 Such view has been described not only in scientific papers and books (Rechenberg, I., Babuska, R., Hadsell, R., Ayub, U., Ikram, A., Shahzad, W., Dhrif, H., Giraldo, L.G., Kubat, M., Wuchty, S. and Erwin, K., 2019, July. Evolution, robotics and the somersaulting spider. In *GECCO'19: Proceedings of the Genetic and Evolutionary Computation Conference* (pp. 99–106). ACM.; Eaton, M., 2015. *Evolutionary Humanoid Robotics*. Springer Berlin Heidelberg) but also in novels (Butler S. Erewhon 1872).

8 For the devastating covid-19 effects (Al-Dadah, O. and Hing, C., 2020. Novel Coronavirus (COVID-19): A Global Pandemic. *The Knee*, *27*(2), p.279.) and the responsibility of the human beings in dangerous virus spill over and diffusion (Johnson, C.K., Hitchens, P.L., Pandit, P.S., Rushmore, J., Evans, T.S., Young, C.C. and Doyle, M.M., 2020. Global shifts in mammalian population trends reveal key predictors of virus spillover risk. *Proceedings of the Royal Society B*, *287*(1924), p.20192736).
